# Effects of ankle-brachial index and brachial-ankle pulse wave velocity on all-cause mortality in a community-based elderly population

**DOI:** 10.3389/fcvm.2022.883651

**Published:** 2022-09-13

**Authors:** Anhang Zhang, Yupeng Liu, Shouyuan Ma, Qiligeer Bao, Jin Sun, Yongkang Su, Shuang Cai, Bokai Cheng, Man Li, Yan Zhang, Tianqi Tao, Jiaojiao Qiu, Jing Dong, Ge Song, Ping Zhu, Shuxia Wang

**Affiliations:** ^1^Medical School of Chinese PLA & Chinese PLA General Hospital, Beijing, China; ^2^Department of Geriatrics, The Second Medical Center & National Clinical Research Center for Geriatric Diseases, Chinese PLA General Hospital, Beijing, China; ^3^Department of Geriatric Cardiology, The Second Medical Center, Chinese PLA General Hospital, Beijing, China; ^4^Department of Outpatient, The First Medical Center, Chinese PLA General Hospital, Beijing, China

**Keywords:** ankle-brachial index (ABI), brachial-ankle pulse wave velocity (baPWV), all-cause mortality, elderly, arterial stiffness

## Abstract

**Background:**

Ankle-brachial index (ABI) and brachial-ankle pulse wave velocity (baPWV) are both important indicators of arterial stiffness and vascular injury. At present, most studies on the relationship between ABI and baPWV and all-cause mortality in community-based elderly are analyzing ABI or baPWV alone, and will focus on a single special population such as diabetes and stroke. The purpose of this study was to evaluate the relationship between ABI and baPWV in a Chinese community-based elderly population, and to analyze their impact on all-cause mortality in a community-based population through a follow-up of nearly 10 years.

**Methods:**

Participants were residents of the Wanshou Road community in Beijing, China. A total of 2,162 people in the community were included, with an average age of 71.48 years. During a mean follow-up period of 9.87 years, 1,826 subjects completed follow-up. Kaplan-Meier survival analysis and different Cox regression models were used to verify the association of ABI and baPWV with all-cause mortality. The selected subjects were divided into two groups according to ABI and baPWV, and ABI was divided into two groups with 0.90 as the cut-off point (group 1: 0.9 < ABI ≤ 1.3; group 2: ABI ≤ 0.9); according to the level of baPWV, they were divided into three groups (Tertile 1: baPWV <1761.5 cm/s; Tertile 2: 1761.5 ≤ baPWV <2121.5 cm/s; Tertile 3: baPWV ≥2121.5 cm/s).

**Results:**

1,826 people were included in the statistical analysis, and the total mortality rate was 181.3/1000. The 10-year all-cause mortality rate of the abnormal ABI group (group 2) was 44.7%, and that of the normal ABI group (group 1) was 17.0%; The 10-year all-cause mortality rates from low to high in the baPWV tertile were 10.0%, 18.7%, and 26.4%. In the Cox proportional hazards model, after adjusting for possible confounders, the effect of baPWV on all-cause mortality was significant, with the 3rd tertile having a 1.647-fold higher risk of all-cause mortality than the 1st tertile (*P* = 0.014 ).

**Conclusions:**

ABI and baPWV are risk factors affecting all-cause mortality in the elderly community population, and baPWV is an independent predictor of all-cause mortality in the elderly community population.

## Introduction

In recent years, much attention has been paid to the role of arterial stiffness in the development of cardiovascular disease (1, 2). Arterial stiffness is now recognized as the most important determinant of elevated systolic and pulse pressures in an aging society and is increasingly used in the clinical assessment of hypertensive patients and various cardiovascular risk factors. Arteriosclerosis is a consequence of increasing age and increases in the context of risk factors for atherosclerosis such as hypertension, diabetes, hypercholesterolemia, and smoking (3).

Arteriosclerosis is one of the pathological conditions of vascular injury and is closely related to atherosclerotic cardiovascular disease. As we all know, pulse wave velocity (PWV) is an indicator of arterial stiffness and a sign of vascular injury (4, 5). Brachial and ankle pulse wave velocity (baPWV) is a new, non-invasive marker of peripheral arteriosclerosis, which has been proven to be an independent predictor of coronary artery disease and all-cause mortality in hypertension and diabetes (6–10). Ankle-brachial blood index (ABI) was originally developed to help diagnose peripheral arterial occlusion disease (PAOD). Currently, ABI <0.9 has been recognized as a reliable sign of peripheral arterial occlusion (11). It is known that patients with ABI <0.9 have a 3–4 times higher risk of cardiovascular death (12). The application of ABI as a predictor of cardiovascular disease has not only been reported in hypertensive populations, but also in some special populations such as hemodialysis patients (13–16).

ABI reflects peripheral arterial stenosis or obstruction, while baPWV can be used to assess the degree of arterial stiffness, both of which are important factors for vascular injury (17). A number of studies have shown that baPWV is a good prognostic predictor for patients with hypertension and diabetes (18–20). Low ABI (<0.9) is an independent predictor of cardiovascular and cerebrovascular events and mortality (21). However, there are few studies that simultaneously measure ABI and baPWV and evaluate their differential clinical value, and there are very few studies on the relationship between ABI, baPWV, and all-cause mortality in elderly community populations. The purpose of this study is to evaluate the relationship between ABI and baPWV in the elderly population in the Chinese community, and to analyze the impact of the two on all-cause deaths in the community population through follow-up in the past 10 years.

## Materials and methods

### Subject

**Figure 1:**
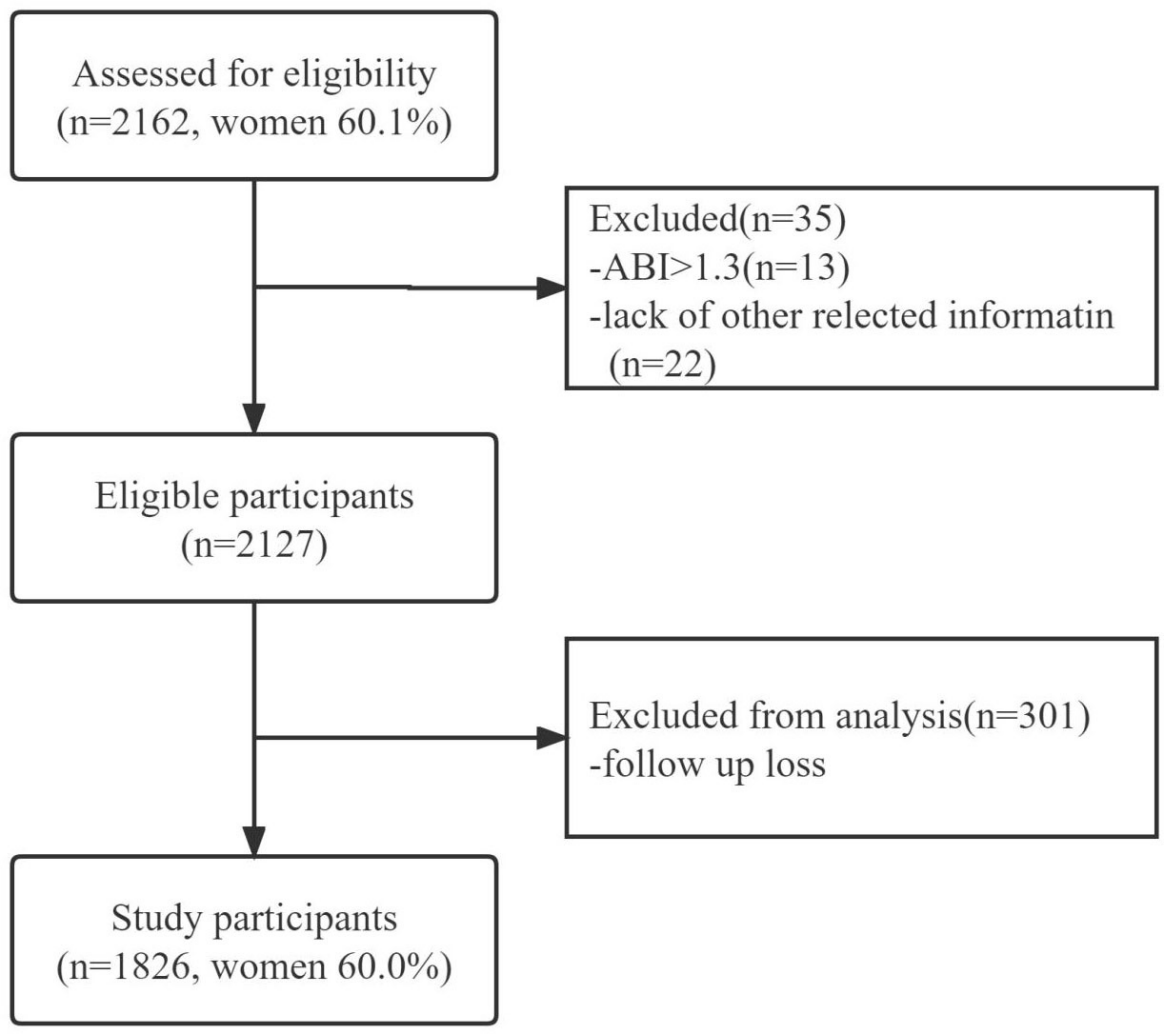
Flow chart for the selection of study participants.

Sampling and research methods have been reported elsewhere (22, 23). The previous study used a two-stage hierarchical cluster sampling method to conduct a population-based cross-sectional survey of participants in Wanshou Road Community, a representative urban residential area in Beijing. This study is a follow-up cohort study based on a previous survey of the community population. The mean age of 2,162 was 71.48 years, the standard deviation was 6.77, and the proportion of females was 60.1%. The Wanshou Road community population was recruited in 2009, and the participants were followed up. Figure 1 shows the recruitment process of the study population. The research program was reviewed and approved by the Ethics Committee of the PLA General Hospital. All participants gave informed consent and identified themselves as Han before being recruited. All investigators were trained at the PLA General Hospital (Beijing, China) and passed the test.

### Data collection

Men and women completed detailed baseline health and lifestyle questionnaires and 2,162 people participated in a health examination by a trained nurse using standard procedures. Alcohol consumption and smoking status, diabetes mellitus, hypertension, history of coronary heart disease (CHD), National Institutes of Health Stroke Scale (NIHSS), Minimum Mental State Examination (MMSE) and Activity of Daily Living (ADL), and other information were obtained from baseline health data and lifestyle questionnaire. Body mass index (BMI) is measured by dividing weight (Kg) by height (m) squared. Height is measured using a separate distance meter and weight is calculated using an electronic scale. After sitting and resting for 5 min, the subjects had their blood pressure measured with a sphygmomanometer. Systolic blood pressure and diastolic blood pressure were measured twice, and the average value was taken for analysis. Hypertension is defined as medically diagnosed hypertension or systolic blood pressure ≥140 mmHg or diastolic blood pressure ≥90 mmHg. Fasting blood glucose, 2-h postprandial blood glucose, glycosylated hemoglobin, serum total cholesterol, high-density lipoprotein cholesterol, low-density lipoprotein cholesterol, and triglyceride were measured by the automatic biochemical analyzer. All field urine samples were collected to determine urinary albumin concentration (mg/ L) and urinary creatinine concentration (g/L), and to calculate UACR (mg/g). All biochemical analyses were performed in the Department of Biochemistry, PLA General Hospital. 1,826 of our subjects agreed to participate in telephone follow-up or signed written informed consent for on-site community follow-up, which constituted our study population. There was no difference in age or sex between respondents and non-responders. This study received ethical approval from the Ethics Committee of the PLA General Hospital.

### ABI and baPWV

**Figure 2:**
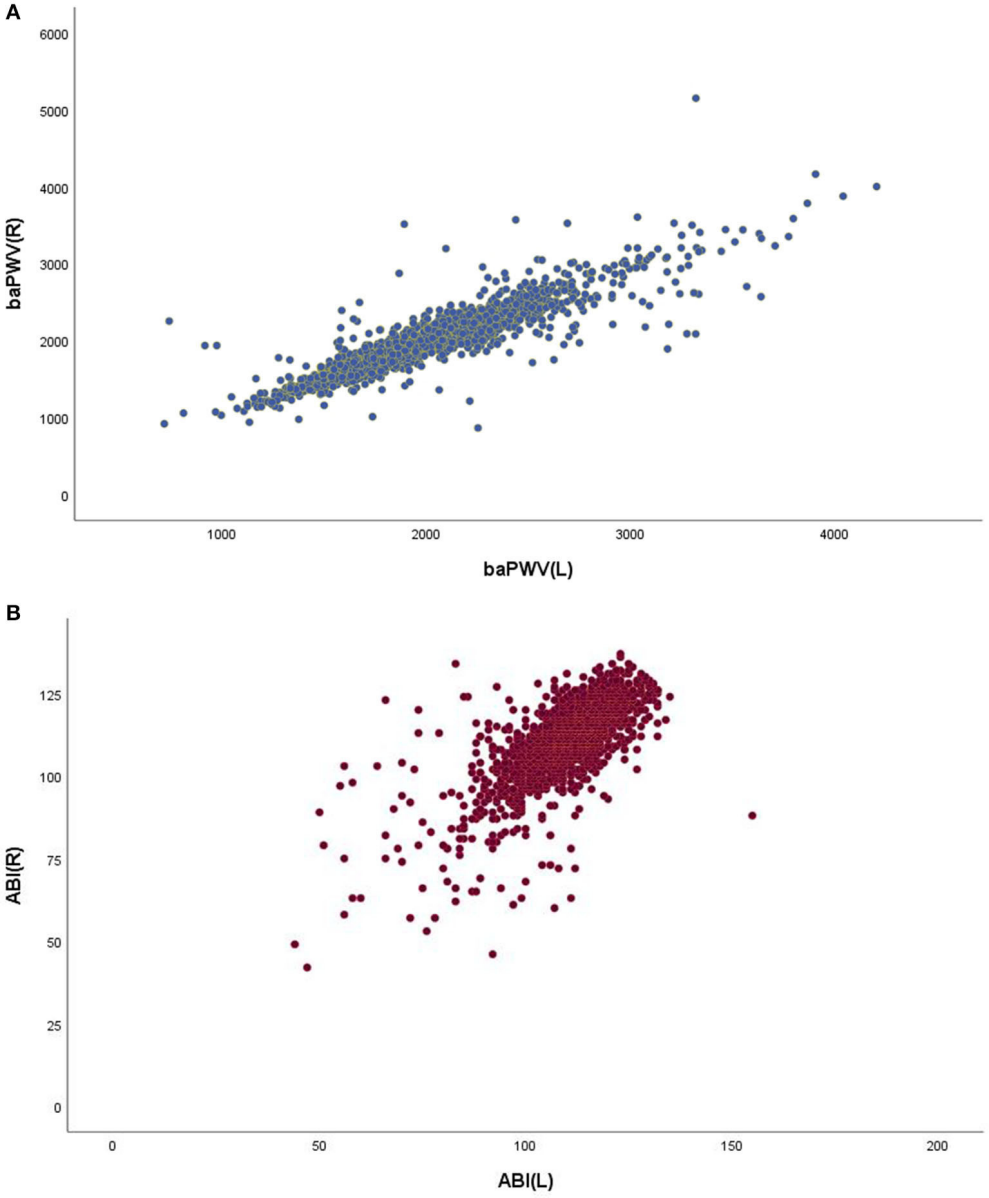
Scatter diagram showing the relationship between the left/right baPWV and the relationship between the left/right ABI.

The values of ABI and baPWV were measured by the VP1000 automatic PWV/ABI analyzer (PWV/ABI, Colin Co., Ltd., Komaki, Japan). ABI is calculated by the ratio of ankle systolic blood pressure divided by arm systolic blood pressure, and is calculated by the lower limit of ankle systolic blood pressure. In order to measure baPWV, the pulse waves obtained from the brachial artery and tibial artery were recorded at the same time, and the transmission time was determined, which was defined as the time interval between the initial increase of waveform of the brachial artery and tibial artery. Calculate the transmission distance from the arm to each ankle joint according to the height. The value of baPWV is automatically calculated as the transmission distance divided by the transmission time. Calculate the average PWV of 7~10 cardiac cycles. Figure 2 shows the relationship between the left and right baPWVs and the left and right ABI. Pearson correlation test showed a significant positive correlation between left and right baPWV (r = 0.905, *P* <0.0001), and a significant positive correlation between left and right ABI (*r* = 0.722, *P* < 0.0001). We used average values of right and left baPWV and left and right ABI in our analysis.

### Groups

Thirteen patients with ABI>1.3 were excluded, and many studies excluded patients with abnormally high ABI, because such ABI may reflect medial artery calcification (MAC). Grouped according to ABI and baPWV, most studies have used dichotomy definition based on ABI critical point 0.90 (24). Similarly, we divided ABI into two groups with 0.90 as the cut-off point. Among the 1,826 (1,096 females and 730 males) study population 1,749 people with ABI ranging from 0.9 to 1.3 were defined as group 1, and 77 people with ABI <0.9 were defined as group 2. Participants were divided into three groups according to baPWV (Tertile1: baPWV <1761.5 cm/s; Tertile 2: 1761.5 ≤ baPWV <2121.5 cm/s; Tertile 3: baPWV≥2121.5 cm/s). A total of 638 people in the 1st third quartile of baPWV were defined as group 1, baPWV <1761.5 cm / S; A total of 604 persons in the second third quartile of baPWV were defined as 21761.5 ≤ baPWV <2121.5 cm/s; A total of 584 people in the third tertile of baPWV were defined as group 3, and baPWV ≥ 2121.5 cm/s.

### Endpoints

Endpoint deaths were defined as indicator events occurring between baseline and follow-up up to December 31, 2020. During follow-up, the primary concern was all-cause mortality. In these analyses, all-cause deaths were defined as deaths from any cause that resulted from phone calls or on-site follow-up provided by family members. For some team members who lost their contact numbers or changed their home addresses, our staff sought help from community workers and police officers in the Population management and Archives Office of Wanshou Road Police Station.

### Statistical analysis of data

All continuous data were expressed as mean ± standard deviation, and categorical data were expressed as absolute value and percentage (%). The Kolmogorov-smirnoff test was used to test whether the data had a normal distribution. The independent sample *T*-test and one-way analysis of variance (ANOVA) were used to analyze the baseline characteristics of the study population. After multiple comparisons, the LSD test is used to assume that the variances are equal, while the Tamhane's T2 test is used to assume that the variances are not equal. The Chi-square test was used to compare all-cause mortality rates after 10 years in different groups. Kaplan-Meier method and log-rank test were used to compare the cumulative probabilities. In Cox proportional risk model analysis, hazard ratio (HR) and 95% confidence interval (CI) were calculated. Model 1: All covariables are not adjusted; Model 2: Adjust age and gender; Model 3: Adjust for age, sex, and ABI or baPWV; Model 4: Adjust for age, gender, height, weight, BMI, waist circumference, systolic pressure, diastolic blood pressure, fasting plasma glucose, postprandial 2 h blood glucose, glycosylated hemoglobin, high-density lipoprotein cholesterol (HDL-C), triglycerides, and serum creatinine, blood uric acid, urine albumin creatinine ratio, smoking history, drinking history, history of CHD, stroke history, NIHSS, MMSE and ADL, a multivariate logistic regression model was established to show the association between ABI and baPWV and all-cause mortality. In cox regression analysis, the Omnibus test was used to test the model coefficients, and the results were analyzed by survival analysis function. In all hypothesis tests, the risk of the first error is prior set at *P* ≥ 0.05. All statistical tests were two-sided and the level of significance was α = 0.05. SPSS software (version 26.0) was used for statistical analysis.

## Results

### Baseline characteristics

In 1826 study populations, independent sample *t*-tests and one-way analysis of variance (ANOVA) tests were used to analyze baseline characteristics of study populations. After the multiple comparisons, the LSD test hypothesis variance is equal, and the Tamhanet2 test hypothesis variance is not equal. Table 1 shows the baseline characteristics of different groupings of people.

**Table 1:** Baseline characteristics of the study population (all men and women), by categories of ABI and baPWV.

**Parameter**	**All patients** **(*n* = 1826)**	**ABI > 0.9** **to ≤ 1.3** ***(n* = 1749)**	**ABI ≤ 0.9** **(*n* = 77)**	**P value**	**Tertile 1 baPWV <1761.5 cm/s (*n* = 638)**	**Tertile 2** **1761.5 ≤ baPWV <2121.5cm/s** **(*n* = 604)**	**Tertile 3** **baPWV ≥2121.5cm/** **(*n* = 584)**	***P*–value**
Age (years)	70.99 ± 7.05	70.77 ± 7.01	76.14 ± 6.01	<0.001	67.37 ± 6.44	71.56 ± 6.60	74.37 ± 6.25	<0.001
Sex (females %)	60.0	60.3	53.2	0.215	57.8	60.8	61.6	0.359
Height (cm)	160.51 ± 8.16	160.57 ± 8.10	159.14 ± 9.30	0.135	162.06 ± 7.95	160.38 ± 7.96	158.95 ± 8.28	<0.001
Weight (kg)	64.49 ± 10.89	64.47 ± 10.78	64.95 ± 13.14	0.754	66.04 ± 10.65	64.66 ± 11.24	62.61 ± 10.51	<0.001
BMI (kg/m^2^)	24.97 ± 3.42	24.95 ± 3.39	25.54 ± 4.06	0.210	25.10 ± 3.39	25.08 ± 3.65	24.71 ± 3.21	0.087
Waist (cm)	88.05 ± 9.31	87.91 ± 9.14	91.38 ± 12.26	0.001	87.66 ± 9.32	88.08 ± 9.74	88.46 ± 8.83	0.328
Hip (cm)	98.21 ± 7.92	98.21 ± 7.73	98.21 ± 11.57	0.997	98.62 ± 7.88	98.15 ± 8.11	97.84 ± 7.76	0.217
Systolic pressure (mmHg)	138.22 ± 19.34	137.69 ± 18.94	150.27 ± 24.02	<0.001	128.06 ± 16.06	139.53 ± 16.73	147.98 ± 19.73	<0.001
Diastolic pressure (mmHg)	77.12 ± 9.74	77.14 ± 9.61	76.78 ± 12.49	0.805	74.14 ± 8.68	76.80 ± 8.96	80.71 ± 10.44	<0.001
FBG (fasting blood glucose) (mmol/L)	6.02 ± 1.51	5.97 ± 1.42	7.19 ± 2.61	<0.001	5.75 ± 1.14	6.02 ± 1.41	6.31 ± 1.87	<0.001
2-h post-meal blood glucose (mmol/L)	8.11 ± 3.17	8.07 ± 3.12	9.33 ± 4.26	0.005	7.64 ± 2.87	8.15 ± 3.23	8.62 ± 3.37	<0.001
Glycosylated hemoglobin (%)	6.07 ± 1.20	6.03 ± 1.10	7.04 ± 2.42	0.001	5.89 ± 0.92	6.05 ± 0.97	6.28 ± 1.58	<0.001
Total cholesterol (mmol/L)	5.25 ± 1.00	5.24 ± 1.00	5.36 ± 1.17	0.386	5.23 ± 0.96	5.23 ± 1.04	5.29 ± 1.02	0.430
High-density lipoprotein (mmol/L)	1.42 ± 0.39	1.42 ± 0.38	1.33 ± 0.48	0.039	1.40 ± 0.38	1.41 ± 0.38	1.44 ± 0.40	0.245
Low-density lipoprotein (mmol/L)	3.23 ± 0.85	3.23 ± 0.85	3.30 ± 0.97	0.445	3.24 ± 0.81	3.21 ± 0.89	3.24 ± 0.87	0.801
Triglyceride (mmol/L)	1.66 ± 0.91	1.65 ± 0.88	1.94 ± 1.28	0.006	1.58 ± 0.73	1.70 ± 1.05	1.71 ± 0.91	0.027
Serum creatinine (umol/L)	74.14 ± 21.21	73.67 ± 21.84	84.80 ± 27.56	0.001	73.15 ± 20.65	74.32 ± 22.03	75.03 ± 23.97	0.327
Blood uric acid (umol/L)	308.45 ± 88.52	307.11 ± 88.09	339.00 ± 93.25	0.002	305.28 ± 90.77	309.69 ± 87.41	310.63 ± 87.20	0.528
UACR	35.91 ± 193.11	34.77 ± 194.96	61.47 ± 143.68	0.235	28.54 ± 200.85	26.20 ± 118.72	54.06 ± 240.05	0.023
NIHSS	0.12 ± 0.55	0.11 ± 0.50	0.45 ± 1.16	0.013	0.09 ± 0.50	0.15 ± 0.63	0.13 ± 0.51	0.166
MMSE	26.96 ± 3.35	27.05 ± 3.21	25.04 ± 5.38	0.002	27.70 ± 2.92	26.94 ± 3.33	26.17 ± 3.63	<0.001
ADL	98.98 ± 5.32	99.03 ± 5.21	97.66 ± 7.19	0.102	99.37 ± 3.14	98.87 ± 5.16	98.65 ± 7.07	0.052
Smoking (%)	30.0	28.7	58.4	<0.001	30.9	31.3	27.6	0.312
Drinking (%)	24.9	24.5	33.8	0.065	28.1	25.5	20.7	0.011
CHD (%)	23.5	22.9	36.4	0.006	19.4	26.5	24.8	0.009
Stroke (%)	12.5	11.9	27.3	<0.001	10.0	14.1	13.7	0.058
ABI	1.10 ± 0.10				1.11 ± 0.11	1.10 ± 0.10	1.11 ± 0.10	0.706
baPWV (cm/s)	1974.16 ± 427.87	1976.52 ± 423.52	1920.42 ± 517.24	0.260				

NIHSS, National Institutes of Health Stroke Scale; MMSE, Minimum Mental State Examination; ADL, Activity of Daily Living; CHD, Coronary Heart Disease.

### Correlation between ABI and baPWV

**Figure 3:**
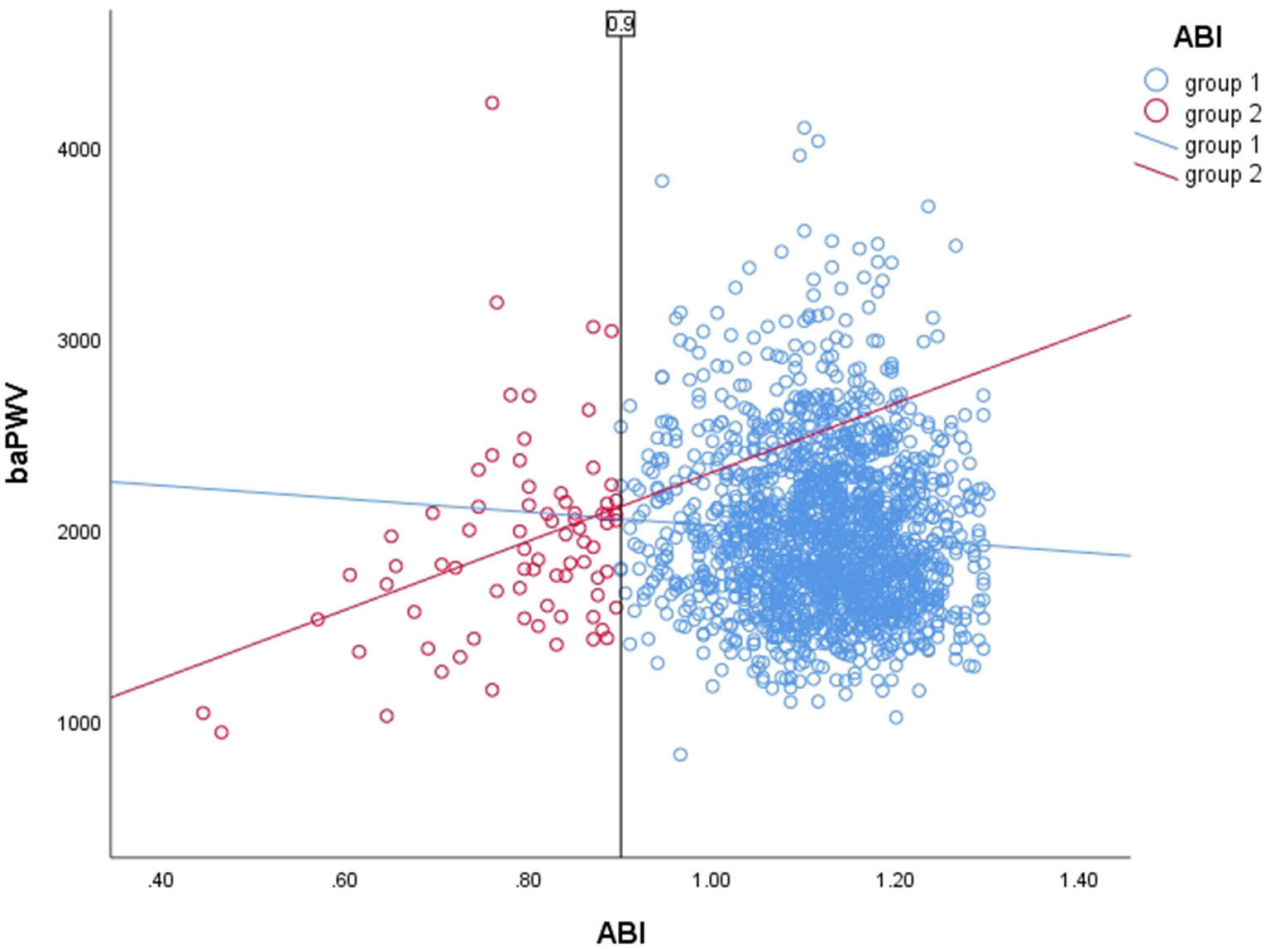
Scatter plot of ABI and baPWV and their regression lines (group 1: R^2^ = 0.004; group 2: R^2^ = 0.113).

Scatter plots of ABI and baPWV and their regression lines show a linear correlation between these two factors at low ABI(<0.9) and high ABI(≥ 0.9), respectively. As shown in Figure 3, this result is similar to what Hayato Matsushima et al. reported in 2017 (25).

### All-cause mortality

During a mean follow-up period of 9.87 years (follow-up 18,029.5 person-years), 1,826 subjects were followed up, of whom 331 died (177 men and 154 women) (Supplementary Files). The total population mortality rate, the crude all-cause mortality rate for men and women are 181.3/1000, 242.5/1000, and 140.5/1000, respectively. Men have a slightly higher mortality rate than women, which is consistent with the results of other studies in the region. Table 2 shows the comparison of all-cause mortality among different subgroups in the chi-square test. As can be seen from Table 2, the 10-year all-cause mortality rate of the abnormal ABI group was 44.7%, much higher than that of the normal ABI group (17.0%), indicating that ABI is an important indicator to roughly judge the all-cause mortality of the elderly in the community. The higher the baPWV was, the higher the all-cause mortality rate was, and the 10-year all-cause mortality rate was 10.0%, 18.7%, 26.4% in the baPWV tripartite group.

**Table 2:** All-cause mortality in different groups.

**Survive Crosstabulation**					
			**Survive**	**All-cause deaths**	**Total**
baPWV	1	Count	574	64	638
group		% within group	90.0%	10.0%	100.0%
	2	Count	491	113	604
		% within group	81.3%	18.7%	100.0%
	3	Count	430	154	584
		% within group	73.6%	26.4%	100.0%
Total		Count	1495	331	1826
		% within group	81.9%	18.1%	100.0%
ABI	1	Count	1453	297	1750
group		% within group	83.0%	17.0%	100.0%
	2	Count	42	34	76
		% within group	55.3%	44.7%	100.0%
Total		Count	1495	331	1826
		% within group	81.9%	18.1%	100.0%

### Mortality risk

**Figure 4:**
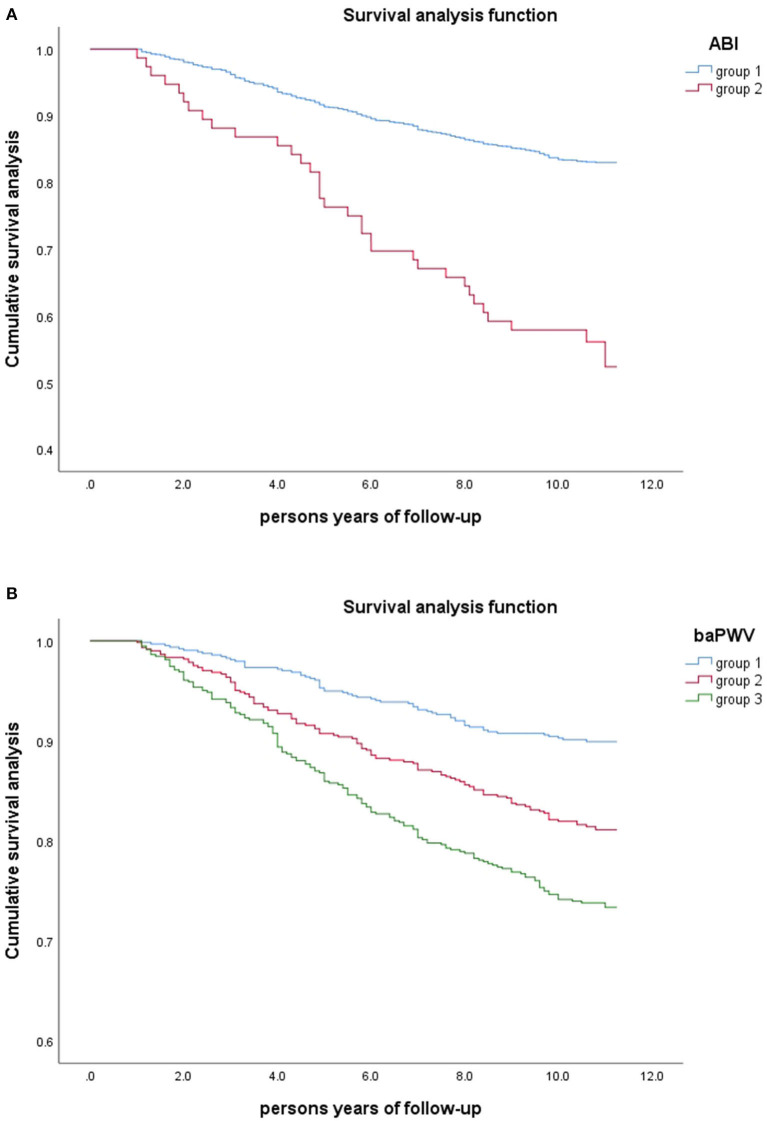
Kaplan-Meier Survival Curves in Different Groups of Study Population (*n* = 1826). **(A)** Grouped by ABI(group 1: 0.9 < ABI ≤ 1.3; group 2: ABI ≤ 0.9). **(B)** Grouped by baPWV (group1: baPWV <1761.5cm/s; group2: 1761.5 ≤ baPWV <2121.5cm/s; group3: baPWV≥2121.5cm/s).

Figure 4 shows the Kaplan Meier survival curve under different ABI and baPWV groups. There was a significant dose-response relationship between ABI, baPWV, and all-cause mortality. With the increase of baPWV value, the survival rate of the study population showed a significant downward trend. And by log-rank test, the *P*–value was <0.001. The cumulative survival risk of the group with abnormal ABI was also greater than that of the group with normal ABI, and the *P*-values were <0.001 after log-rank tests.

### COX survival analysis

To investigate the effects of ABI and baPWV on all-cause mortality, we performed Cox proportional risk model fitting on the data. We built four Cox proportional risk models. In addition to ABI and baPWV, the following variables were included in the regression analysis that could be used to explain all-cause death and inter-group differences in the baseline analysis: age, gender, height, weight, BMI, waist circumference, systolic blood pressure, diastolic blood pressure, fasting blood glucose, 2-h postprandial blood glucose, glycosylated hemoglobin, high-density lipoprotein, triglyceride, blood creatinine, blood uric acid, urinary albumin creatinine ratio, smoking history, drinking history, coronary heart disease history, stroke history, NIHSS, MMSE, ADL.

As shown in Table 3, in model 1 without adjusting for any covariates, the risk of all-cause death was 3.144 times higher in the abnormal ABI group than in the normal group. After adjusting for age and sex, the risk of all-cause death in model 2 decreased in the abnormal ABI group by 1.799 times that in the normal group. After adjusting for age, sex, and baPWV, the risk of all-cause death was 1.975 times higher in the abnormal ABI group than in the normal group in model 3, which was higher than in model 2. In model 4, there was no statistically significant difference in the risk of all-cause death between the abnormal ABI group and the normal ABI group after adjusting for all possible covariates affecting survival.

**Table 3:** Cox regression analysis of the relationship between ABI, baPWV, and all-cause mortality.

		**HR(95%CI)**			
		**Model 1**	**Model 2**	**Model 3**	**Model 4**
ABI	group 1	1 (Reference)	1 (Reference)	1 (Reference)	1 (Reference)
	group 2	3.144*** (2.204,4.484)	1.799** (1.251,2.585)	1.975*** (1.368,2.851)	1.069 (0.596,1.916)
baPWV	group 1	1 (Reference)	1 (Reference)	1 (Reference)	1 (Reference)
	group 2	1.959*** (1.441,2.661)	1.289 (0.938,1.771)	1.362 (0.990,1.874)	1.215 (0.829.1.780)
	group 3	2.905*** (2.170,3.888)	1.504** (1.101,2.053)	1.707*** (1.244,2.341)	1.647* (1.106,2.452)
	P for trend	<0.001	0.010	0.002	0.008

HR, hazard ratio; CI, confidence interval.

^*^P <0.05.

^**^P < 0.01.

^***^P < 0.001.

In the analysis of the association between baPWV and all-cause mortality, in model 1, the risk of all-cause mortality was 1.959 and 2.905 times for the 2nd and 3rd tertile groups (group 2 and group 3) compared with baPWV 1st trine (group 1) without adjusting for any covariate. After adjusting for age and sex, in model 2, the risk of all-cause death in group 2 and group 3 was 1.289 and 1.504 times higher than that in group 1, using group 1 as a reference. After adjusting for age, sex, and ABI, in model 3, the risk of all-cause death in group 2 and 3 was 1.362 and 1.647 times higher than in group 1, using group 1 as a reference. In model 4, after adjusting all covariates that may affect survival, and taking group 1 as a reference, there was no statistically significant difference in all-cause mortality of group 2 and group 1, and the risk of all-cause death of group 3 was 1.647 times that of group 1, the difference was statistically significant (*P* = 0.014). BaPWV is an independent risk factor for predicting all-cause mortality.

**Figure 5:**
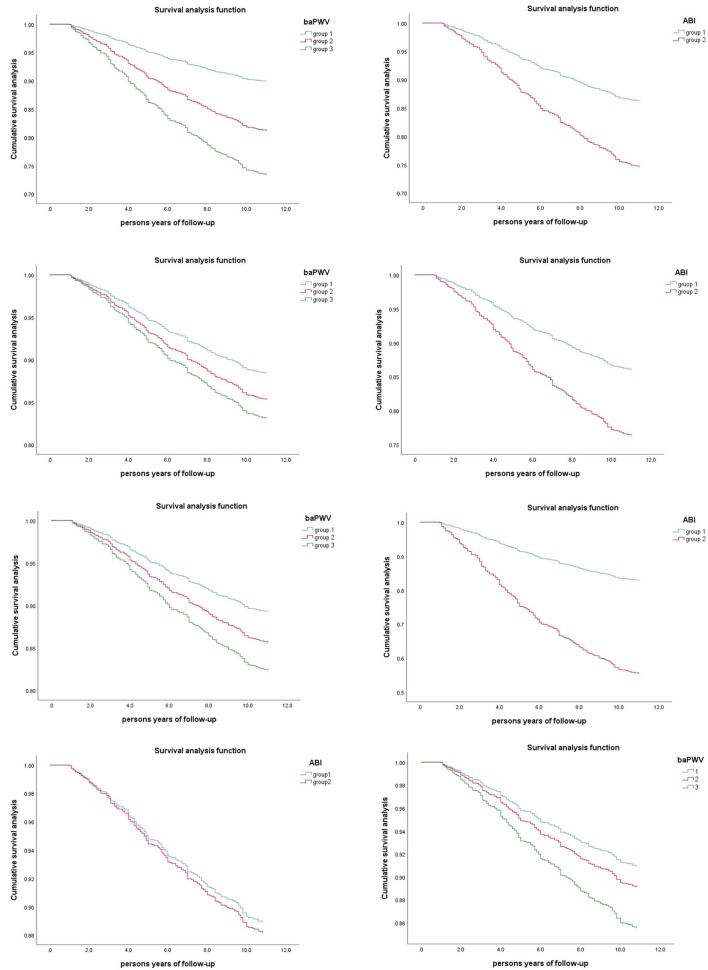
Survival analysis curves were drawn for the four models of ABI and baPWV in the Cox proportional risk model.

As shown in Figure 5, survival analysis curves were drawn for the four models of ABI and baPWV in the Cox proportional risk model. Survival analysis curves for the four models also showed significant differences in survival rates among ABI and baPWV subgroups. Ten-year all-cause mortality was significantly higher in the abnormal ABI group than in the normal ABI group. The greater the baPWV, the greater the risk of death within the group.

**Figure 6:**
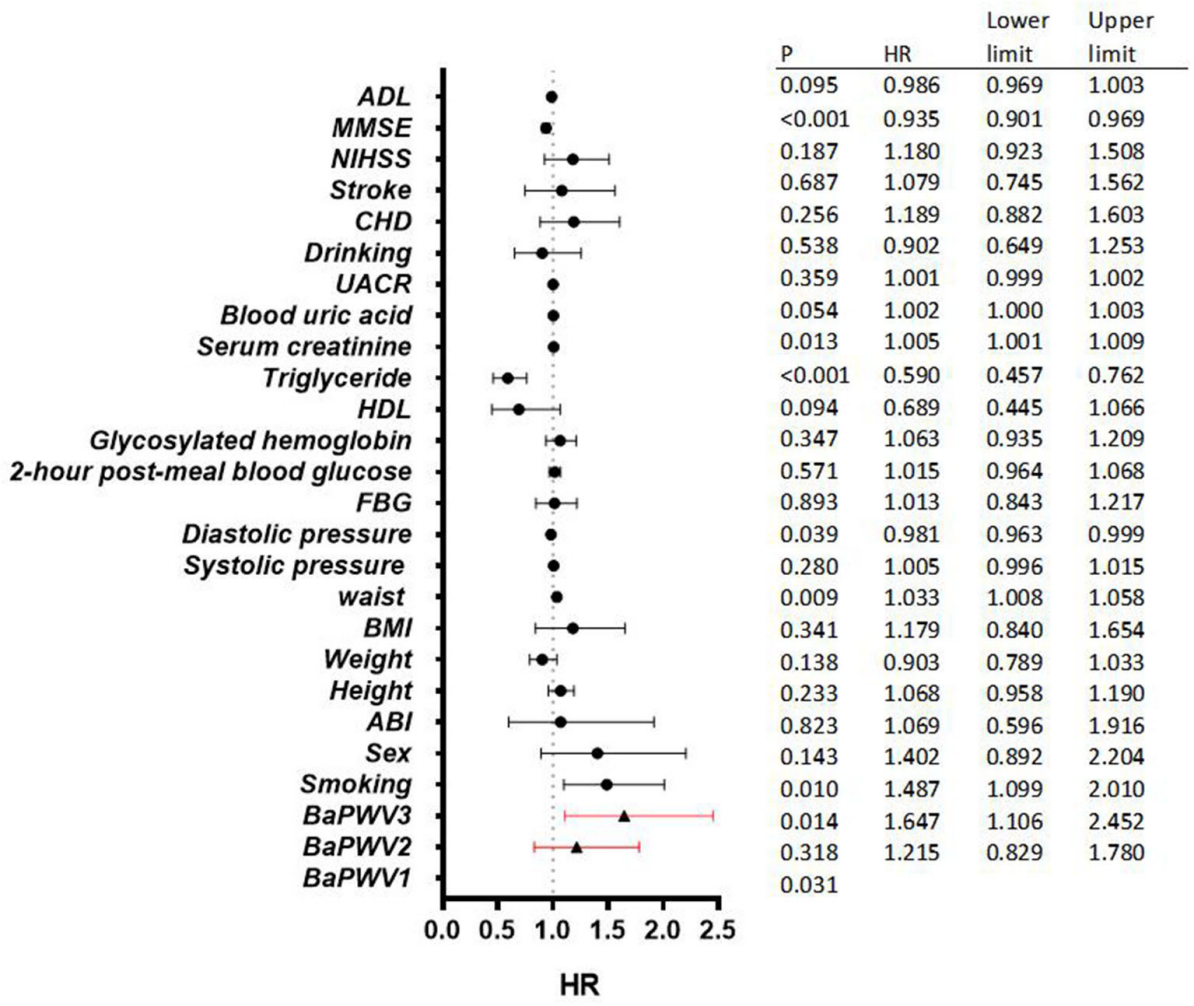
Forest plot for each covariate in the cox regression model.

As can be seen from the forest plot in Figure 6, the results of the forest plot use multivariate cox regression analysis, and our forest plot is a display of the results of variables in the Cox regression model 4. Among the variables affecting all-cause mortality in the multivariate Cox regression model, baPWV had a significant effect on all-cause mortality, and the risk of all-cause mortality in the third quartile was 1.647 times higher than that in the first quartile (*P* = 0.014). Compared with other covariables in the forest plot, baPWV had the highest risk of all-cause mortality. Not included in the model of our forest Figure 4 of the variables of the age, because of our study population as the community elderly people over the age of 60, the growth of the age and natural aging in risk for all-cause mortality is larger, the higher the HR value influence diagram shows the result of the forest, we didn't show age figure the result in the forest, but in the Cox regression model, Age was included in the regression analysis as a covariate.

### Hierarchical analysis

**Figure 7:**
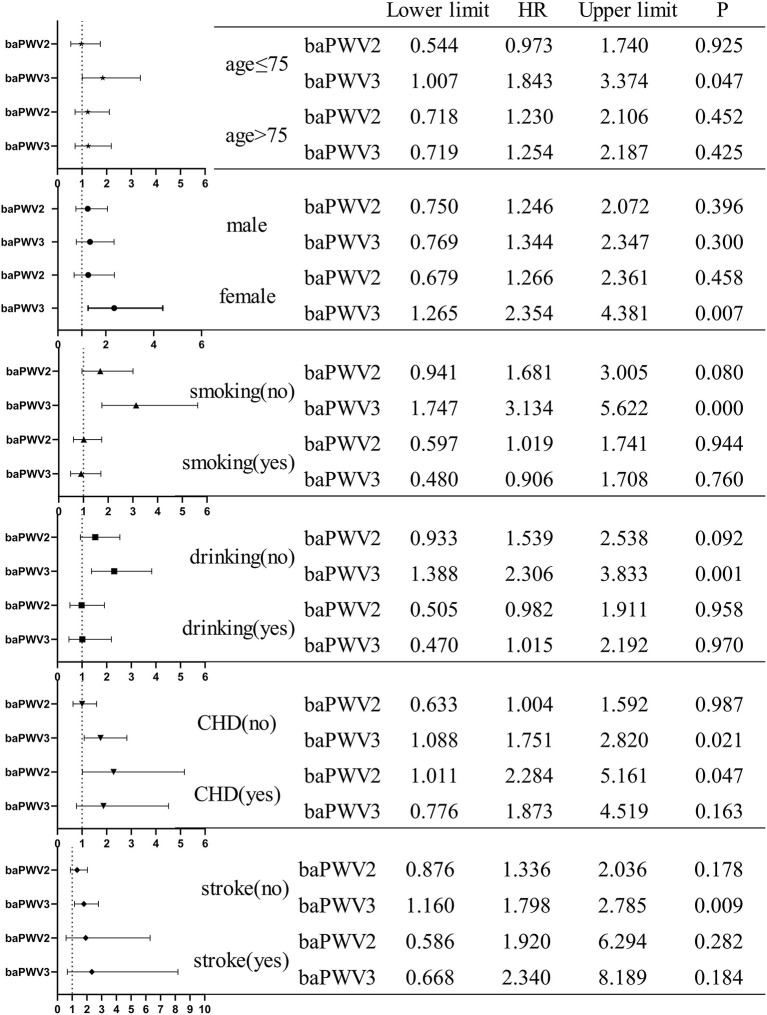
Forest map with stratified analysis by age, sex, smoking history, drinking history, history of coronary heart disease, and history of stroke.

Hierarchical analysis was performed for covariates with greater influence on all-cause mortality in the Cox risk model, stratified by age, sex, history of smoking, history of alcohol consumption, history of coronary heart disease, history of stroke, and ABI. After controlling for all covariates except stratified variables, the association between baPWV and risk of all-cause death persisted in the subgroup analysis. Figure 7 shows a hierarchical forest plot showing a statistically significant association between baPWV 3rd tertile and all-cause death among individuals younger than 75 years of age, women, and those with no history of smoking, alcohol consumption, coronary heart disease, and stroke.

**Figure 8:**
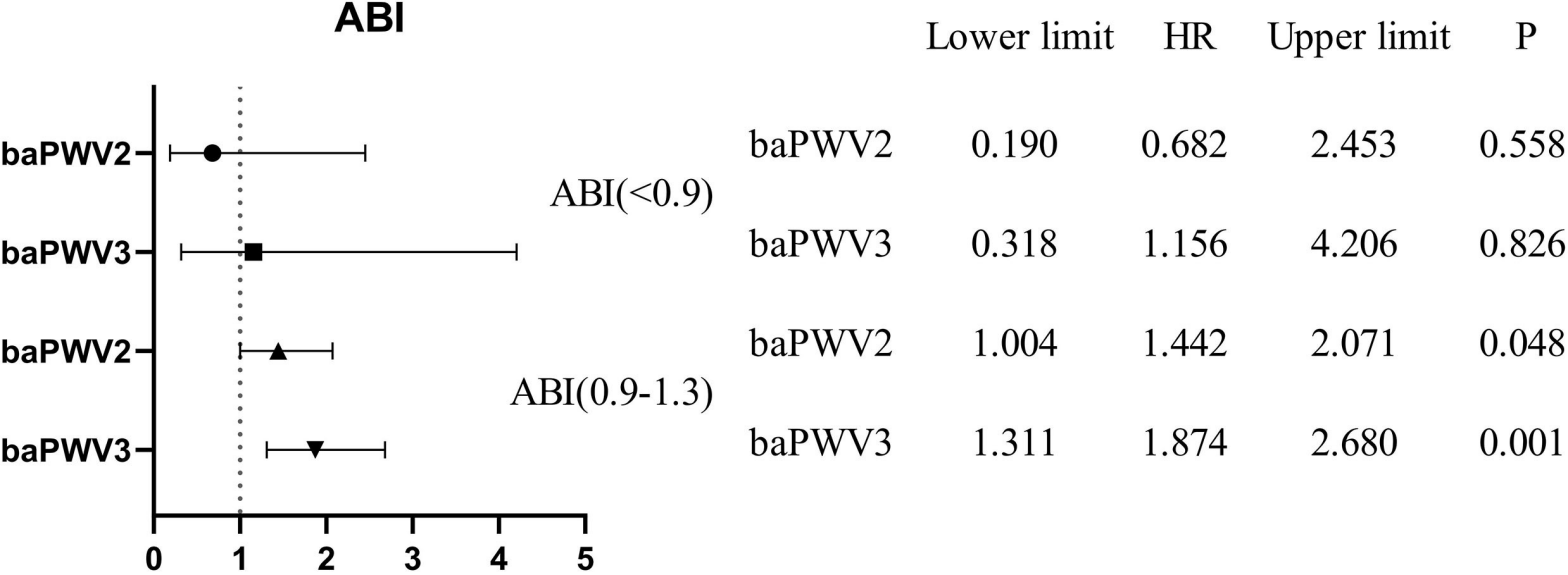
Hierarchical forest map of the ABI.

As shown in Figure 8, the results of stratification of the ABI showed that the all-cause mortality rate in the second and third quintiles of baPWV was 1.442 and 1.874 times higher than that in the first quintile of baPWV (*P* = 0.048, *P* = 0.001) in those with an ABI in the normal range (0.9–1.3).

## Discussion

From our results, it appears that ABI is a risk factor for all-cause mortality, but not an independent risk factor, and that it is influenced by other covariates. ABI can be used as a preliminary predictor of all-cause mortality. Which did not adjust for any covariates, the risk of all-cause mortality in the abnormal ABI group was 3.144 times higher than in the normal group. After adjusting for all relevant covariates that may affect survival, the difference in risk of all-cause mortality between the abnormal ABI and normal ABI groups was not statistically significant. Our results show that baPWV is an independent risk factor for all-cause mortality. Compared with the group 1, without adjustment for any covariates, baPWV was 1.959 times and 2.905 times more likely to be associated with risk of all-cause death in group 2 and group 3. After adjusting for all relevant covariates that may affect survival, the risk of all-cause death in group 3 was 1.647 times higher than in group 1, a statistically significant difference (*p* = 0.014). ABI can be used first as a primary screening indicator for vascular injury, but even if the ABI is normal, vigilance should still not be relaxed and the vascular status of the elderly can then be determined by baPWV. From our ABI stratification results, for the abnormal ABI group, with group 1 as the reference, the HR in Cox regression for Group 3 was 1.156. For those with an ABI in the normal range (0.9–1.3), all-cause mortality was 1.442 and 1.874 times higher in baPWV tertile 2 and 3 than in baPWV tertile 1. From the results of the correlation analysis, it is known that baPWV is strongly correlated with ABI and that the lower HR in the abnormal ABI group may be due to the fact that the abnormal ABI all-cause mortality rate was 44.7% and the higher all-cause mortality rate had an impact on the baPWV results.

There are no previous exhaustive studies on the relationship between ABI and baPWV and all-cause mortality in older people in the community. Most studies have analyzed ABI or baPWV separately, as well as focusing on a single specific population such as diabetes, stroke, etc. Yasutaka Maeda et al. reported in a 2014 study that baPWV predicted all-cause mortality and cardiovascular events in patients with diabetes (26). They included 3,628 subjects, prospectively followed for 3.2 to 2.2 years, and demonstrated that high baPWV was a useful independent predictor of mortality and cardiovascular morbidity in patients with diabetes. Compared to their study, our study performed a stratified analysis of ABI and we had a longer mean follow-up of 9.87 years. The REBOUND study by Jeong Mi Kim et al. in 2020 also indicated that arterial stiffness is an independent predictor of mortality risk in patients with type 2 diabetes (27). During a mean follow-up period of 8.6 years, there were 199 deaths (8.6%) in the study population. Compared to their study, our population had an all-cause mortality rate of 18.1%, much higher than the REBOUND study population. This reflects the particularity of our study population. The community elderly population we selected has the characteristics of advanced age and multiple diseases. The analysis of a special population of a single disease may not be applicable to the community elderly population. In 2014, Li-Hsin Chang et al. mentioned in their study in Taipei that the combination of ankle-brachial index and brachial-ankle pulse wave velocity was better correlated with prognosis in diabetic patients (28). It is this study that provides new ideas for our research, considering the relationship between ABI and baPWV, and their impact on all-cause mortality. In 2010, the Takashima study done in Japan by Tanvir Chowdhury Turin et al. found that baPWV predicted all-cause mortality in the general population with a follow-up of 6.5 years (29). Participants in the highest tertile of baPWV had an increased risk of all-cause mortality compared to those in the lowest tertile, with a multivariable-adjusted hazard ratio of 6.8 (95% confidence interval: 1.4–32.8). Our study differs from theirs in that the study population is different; our population is older people aged >60 years, who already have a lower life expectancy than younger people, and the bias introduced by age is heavy, resulting in a lower HR in our study than in theirs. In 2010, a study by Ichiro Miyano et al. examined the relationship between baPWV and 3-year mortality in community-dwelling older adults (30). Their study also took note of a specific population of elderly people in the community. Compared to their study, we had a longer follow-up period, a larger included population and more detailed groupings of baPWV and ABI. Therefore, our results may be particularly convincing.

In addition, there are other studies on the relationship between all-cause mortality and ABI and baPWV in specific populations. In 2014, a study by Jinkwon Kim et al. found that baPWV was a strong predictor of mortality in patients with acute stroke (31). In 2016, a study by Kazuki Ikura et al. found that baPWV rather than ABI predicted all-cause mortality after lower limb amputation (LEA) in diabetic patients (32). In 2005, Tokuyuki Kitahara et al. studied the effects of baPWV and ABI on morbidity and mortality in hemodialysis patients and showed that ABI, but not baPWV, was a strong predictor of mortality in hemodialysis patients (11). However, baPWV is useful when selecting high-risk groups in patients with an ABI > 0.9. Therefore, screening hemodialysis patients by baPWV and ABI may provide additional information for identifying high-risk groups. In 2011, a study by Miho Tanaka et al. found that ankle-brachial index, but not brachial-ankle pulse wave velocity, was a strong predictor of morbidity and mortality from systemic atherosclerosis in maintenance hemodialysis patients (13).

Given the previous studies, we can find that ABI and baPWV have an effect on all-cause mortality, and although the extent of the effect on all-cause mortality varies in the outcome, the predictive role of ABI and baPWV on all-cause mortality can be established. By comparing our study with other recent or well-known studies, we can identify several advantages. Our population is a community-based elderly population, a group that is currently, and will be in the future, under great medical pressure as the aging process increases. The study of all-cause mortality in the elderly population has important implications for the prevention of disease in the elderly population. In addition, the long follow-up period of our study, averaging 9.87 years (follow-up 18,029.5 person-years), lends credence to our results.

Age, increased inflammatory activity, oxidative stress, endothelial dysfunction, and peripheral arteriosclerosis may underlie abnormal ABI and baPWV in these older adults (1, 23, 31). Determinants of arterial stiffness are the effects of smooth muscle cells and inflammation. For many years, research into the molecular determinants of arterial stiffness has focused on the structure and quantity of the main load-carrying proteins: elastin and collagen (33). Indeed, aging and blood pressure, the two major determinants of arterial stiffness, are associated with many molecular changes in elastic arterial load-bearing mediators. The ordered arrangement of elastic fibers and sheets was gradually observed to thin, split, wear and chip over time. Degeneration of elastic fibers is associated with an increase in collagenous material and matrix, usually accompanied by calcium deposition in the matrix and degenerated elastic fibers (34). Chronic inflammatory processes have also been reported to harden large arteries. This may occur through a variety of mechanisms, including endothelial dysfunction, the cellular release of numerous inducible matrix metalloproteinases (including matrix metalloproteinase [MMP]-9), medial calcification, changes in proteoglycan composition and hydration status, and perivascular Cell infiltration, leading to vascular ischemia (3).

The pulse wave propagates along the arterial wall, reflecting the arterial function, which is mainly related to the compliance of the blood vessel. The faster the baPWV, the worse the compliance and the higher the stiffness of the artery. ABI reflects the arterial structure and is related to the opening of peripheral arteries. ABI <0.9 indicates that the blood flow of the diseased blood vessels is reduced to varying degrees (35). Our stratified analysis findings also suggest that higher baPWV may be associated with higher all-cause mortality among community-dwelling, well-functioning older adults with a history of cardiovascular, coronary heart disease, or stroke.

## Conclusions

ABI and baPWV measurements are widely used and the results are reliable and reproducible compared to other non-invasive automated procedures. ABI and baPWV are risk factors affecting all-cause mortality in the elderly community population, and baPWV is an independent predictor of all-cause mortality in the elderly community population. Given the simplicity of this technology and its possible predictive value for independent future mortality in a world facing an aging world today, ABI and baPWV would be ideal for large-scale population screening and incorporation into routine clinical settings. Efforts to prevent age-related arteriosclerosis through ABI and baPWV screening may improve the health of older adults.

## Data availability statement

The raw data supporting the conclusions of this article will be made available by the authors, without undue reservation.

## Ethics statement

Written informed consent was obtained from the individual(s) for the publication of any potentially identifiable images or data included in this article.

## Author contributions

AZ, YL, and SM proposed the design of the study and wrote the main manuscript text. QB, JS, YS, SC, and ML organize data and analyze data. BC, TT, JQ, GS, and YZ prepared figures and tables. SW and PZ corrected the manuscript. All authors reviewed the manuscript.

## Funding

This study was supported by the National Key R&D Program of China (Funding No. 2020YFC2008900), the Military Medical Youth Growth Project of PLA General Hospital (Funding No. QNC19005), the Logistics Scientific Research Project of the Chinese PLA (Funding No. 19BJZ30), and the National Defense Science and Technology Innovation Special Zone Project (19-163-15-ZD-009-001-10).

## Conflict of interest

The authors declare that the research was conducted in the absence of any commercial or financial relationships that could be construed as a potential conflict of interest.

## Publisher's note

All claims expressed in this article are solely those of the authors and do not necessarily represent those of their affiliated organizations, or those of the publisher, the editors and the reviewers. Any product that may be evaluated in this article, or claim that may be made by its manufacturer, is not guaranteed or endorsed by the publisher.

## Abbreviations

ABI: ankle-brachial index
baPWV: brachial-ankle pulse wave velocity
PWV: pulse wave velocity
PAOD: peripheral arterial occlusive disease
MAC: medial artery calcification
HDL-C: high-density lipoprotein cholesterol
HR: hazard ratio
CI: confidence interval
NIHSS: National Institutes of Health Stroke Scale
MMSE: Minimum Mental State Examination
ADL: Activity of Daily Living
CHD: Coronary Heart Disease
LEA: lower limb amputation
MMP: matrix metalloproteinase.
