# Supplementary material for: Effects of ankle-brachial index and brachial-ankle pulse wave velocity on all-cause mortality in a community-based elderly population
Source: Front Cardiovasc Med. 2022 Sep 13;9:883651. doi: 10.3389/fcvm.2022.883651 (PMC9513615; doi:10.3389/fcvm.2022.883651)
Supplement: Supplementary file 1 [file Data_Sheet_1.docx]

Table S1: The number and proportion of each cause of death in the death population.

| Cause of death | Multimorbidity (hypertension, diabetes, coronary heart disease, etc.) | Myocardial infarction, heart failure | Cancer | Pneumonia | Multiple organ failure | Stroke | Trauma | Unexplained death | Total |
| --- | --- | --- | --- | --- | --- | --- | --- | --- | --- |
| Number of cases | 142 | 61 | 51 | 16 | 29 | 12 | 4 | 16 | 331 |
| % of total | 42.90% | 18.43% | 15.41% | 4.83% | 8.76% | 3.63% | 1.21% | 4.83% | 100% |

Multimorbidity: Multimorbidity is the presence of two or more long-term conditions, which refers to the coexistence of multiple diseases and clinical problems, and is characteristic of the elderly.
